# Supplementary material for: Fluid face but not gender: Enfacement illusion through digital face filters does not affect gender identity
Source: PLoS One. 2024 Apr 3;19(4):e0295342. doi: 10.1371/journal.pone.0295342 (PMC10990241; doi:10.1371/journal.pone.0295342)
Supplement: S1 File — (DOCX) [file pone.0295342.s001.docx]

# Supporting Information

#

# S1 Appendix. Supplementary Tables

**S1 Table. Results for Homogeneity of Variance and Normality Testing of Illusion Questionnaire and M-F-VAS**

| **Dependent Variable** | **Independent Variable** | **Levene’s Test** | **Shapiro-Wilk-Normality-Test** |
| --- | --- | --- | --- |
| Illusion Rating | synchrony gender | *F*1, 134 = 0.24, p = 0.625  *F*1, 134 = 0.139, p = 0.710 | *W* = 0.978, p = 0.026^**^ |
| Control item | synchrony gender | *F*1, 134 = 0.116, p = 0.734  *F*1, 134 = 0.678, p = 0.412 | *W* = 0.943, *P* < 0.001^***^ |
| Agency | synchrony gender | *F*1, 134 = 4.592, p = 0.034^*^  *F*1, 134 = 0.556, p = 0.457 | *W* = 0.924, *P* < 0.001^***^ |
| Ownership | synchrony gender | *F*1, 134 = 10.147, p = 0.002^**^  *F*1, 134 = 0.29, p = 0.591 | *W* = 0.96, *P* < 0.001^***^ |
| Attractiveness | synchrony gender | *F*1, 134 = 0.438, p = 0.509  *F*1, 134 = 0.318, p = 0.574 | *W* = 0.98, *P* = 0.039^*^ |
| Similarity | synchrony gender | *F*1, 134 = 0.861, p = 0.355  *F*1, 134 = 0.106, p = 0.745 | *W* = 0.942, *P* < 0.001^***^ |
| Mood | synchrony gender | *F*1, 134 = 0.984, p = 0.323  *F*1, 134 = 0.013, p = 0.91 | *W* = 0.94, *P* < 0.001^***^ |
| M-F-VAS | synchrony gender | *F2*, 201 = 7.022; *P* = 0.001^**^  *F1*, 202 = 3.904; *P* = 0.05^*^ | *W* = 0.971, *P* < 0.001^***^ |

Level of significance: *: p≤ 0.05; **: p≤ 0.01; ***: p≤ 0.001

Illusion Questionnaire consisted in two items (ownership + agency). Masculinity-femininity-rating and Illusion questions were both rated on a visual analogue scale (VAS).

**S2 Table. Results of the nonparametric ART-ANOVAs performed (Illusion Questionnaire and M-F-VAS)**

| **Dependent Variable** | **Within-Factor: synchrony** | **Between-Factor: gender** | **Interaction: synchrony x gender** |
| --- | --- | --- | --- |
| Illusion Rating | *F*1, 66 = 24,36; *p* < 0.001^***^, η^2^ = 0.27 | *F*1, 66 = 0.196; *p* = 0.66, η^2^= 0.003 | *F*1, 66 = 24,36; *p* < 0.001^***^, η^2^ = 0.27 |
| Control item | *F*1, 66 = 0.175; *p* = 0.677, η^2^ = 0.003 | *F*1, 66 = 0.445; *p* = 0.507, η^2^ = 0.003 | *F*1, 66 = 0.367; *p* = 0.547, η^2^= 0.006 |
| Agency | *F*1, 66 = 21.33; *p* < 0.001^***;^ η^2^=0.244 | *F*1, 66 = 0.522; *p* = 0.473, η^2^ = 0.008 | *F*1, 66 = 1.526; *p* = 0.221, η^2^ = 0.023 |
| Ownership | *F*1, 66 = 7.989; *p* = 0.006^**^, η^2^= 0.108 | *F*1, 66 = 0.007; *p* = 0.935, η^2^ < 0.001 | *F*1, 134 = 0.004, p = 0.951, η^2^ < 0.001 |
| Attractiveness | *F*1, 66 = 0.605; *p* = 0.439, η^2^= 0.009 | *F*1, 66 = 5.09; *p* = 0.027^*^, η^2^= 0.072 | *F*1, 66 = 0.101; *p* = 0.752, η^2^= 0.002 |
| Similarity | *F*1, 66 = 6,78; *p* = 0.011^*^, η^2^= 0.093 | *F*1, 66 = 0.331; *p* = 0.567, η^2^= 0.005 | *F*1, 66 = 3.810; *p* = 0.055, η^2^= 0.055 |
| Mood | *F*1, 66 = 6,78; *p* = 0.093, η^2^= 0.093 | *F*1, 66 = 3,134; *p* = 0.081, η^2^= 0.045 | *F*1, 66 = 2,008; *p* = 0.161, η^2^= 0.03 |
| M-F-VAS | *F2*, 132 = 97.86; *p* < 0.001^***^, η^2^= 0.597 | *F1*, 66 = 2.33; *p* = 0.131, η^2^= 0.034 | *F2*, 132 = 6,92; *p* = 0.001^**^, η^2^= 0.095 |

Level of significance: *: p≤ 0.05; **: p≤ 0.01; ***: p≤ 0.001

Illusion Questionnaire consisted in two items (ownership + agency). Masculinity-femininity-rating and Illusion questions were both rated on a visual analogue scale (VAS). Analyses were performed using aligned ranks transformation analysis of variance (ANOVA) for nonparametric factorial analyses (ART–ANOVA; Wobbrock et al., 2011).

**S3 Table. Model comparison for the analysis with linear mixed models of the IAT-Data**

| **Model comparison** | **Full model** | **AIC** | **BIC** | **Chisq** | **df** | **P** |
| --- | --- | --- | --- | --- | --- | --- |
| Model_1c | M_1c: RT ~ cong + (1 \| subject) + (1 \| item) | -7622.8 | -7593 |  |  |  |
| Model_2a vs. Model_1c | M_2a: RT ~ cong + (1\| ID) + (1\| item) | -7692.2 | -7655 | 71.416 | 1 | <0.001^***^ |
| Model_2b vs. Model_1c | M_2b: RT ~ synch + (1\| ID) + (1\| item) | -7643.1 | -7605.9 | 22.316 | 1 | <0.001^***^ |
| Model_3a vs. Model_2a | M_3a: RT ~ cong + synch + (1\| ID) + (1\| item) | -7712.4 | -7667.8 | 22.238 | 1 | <0.001^***^ |
| Model_3b vs. Model_3a | M_3b: RT ~ cong * synch + (1\| ID) + (1\| item) | -7711.5 | -7659.4 | 1.111 | 2 | 0.292 |
| Model_3c vs. Model_1b | M_3c: RT ~ cong * illusion + (1\| ID) + (1\| item) | 7688.8 | -7636.7 | 0.582 | 2 | 0.747 |
| Model_4 vs. Model_3a | M_4: RT ~ cong + synch + (1 + cong* synch\| ID)  + (1\| item) | -8511.5 | -8347.8 | 817.07 | 1 | <0.001^***^ |

Level of significance: *: p≤ 0.05; **: p≤ 0.01; ***: p≤ 0.001

Abbreviations in alphabetical order: **AIC** – Akaike information criterion; **BIC** – Bayesian-Information-Criterion; **cong** – factor with two levels: congruent vs. incongruent; **df** – degrees of freedom; **IAT** – Implicit Association Test; **ID** – participants; **illusion** – Illusion score: difference of the illusion ratings (agency + ownership) between the synchronous and asynchronous condition; **item** – words presented during the IAT;**RT** – reaction times; **synch** – factor with two levels: synchronous vs. asynchronous.

**S4 Table. Effect sizes in Confidence Intervals of IAT-Data analysis with linear mixed models**

| **Model** | **Full model** | **Effect** | **b** | **SE** | **df** | **t** | **P** | **CI-l** | **Cl-u** |
| --- | --- | --- | --- | --- | --- | --- | --- | --- | --- |
| Model_1a | RT ~ 1 + (1 \| ID) | intercept | 6.929 | 0.012 | 63.826 | 577.4 | < 0.001^***^ | 6.905 | 6.953 |
| Model_1b | RT ~ 1 + (1 \| item) | intercept | 6.918 | 0.009 | 19.028 | 805.1 | < 0.001^***^ | 6.901 | 6.936 |
| Model_1c | RT ~ 1 + (1 \| item) + (1 \| ID) | intercept | 6.931 | 0.015 | 74.256 | 462.7 | < 0.001^***^ | 6.901 | 6.961 |
| Model_2a | RT ~ cong + (1 \| ID) + (1 \| item) | congruency | 0.027 | 0.003 | 12540 | 8.463 | < 0.001^***^ | 0.020 | 0.033 |
| Model_2b | RT ~ synch + (1 \| ID) + (1 \| item) | synchrony | -0.015 | 0.003 | 12540 | -4.726 | < 0.001^***^ | -0.021 | -0.009 |
| Model 3a | RT~ cong + synch + (1 \| ID) + (1 \| item) | congruency synchrony | 0.027  -0.015 | 0.003  0.003 | 12540  12540 | 8.463  -4.726 | < 0.001^***^  < 0.001^***^ | 0.020  -0.021 | 0.033  -0.009 |
| Model_3b | RT ~ congruency * synch + (1 \| ID) + (1 \| item) | congruency synchrony cond. * cong. | 0.023  -0.018  0.007 | 0.004  0.004  0.006 | 12540  12540  12540 | 5.188  -4.118  1.054 | < 0.001^***^  < 0.001^***^ 0.292 | 0.014  -0.027  -0.006 | 0.032  -0.009  0.019 |
| Model_3c | RT ~ cong * illusion + (1 \| ID) + (1 \| item) | illusion congruency ill. * cong. | -0.004  0.028  -0.006 | 0.031  0.004  0.008 | 65.071  12540  12540 | -0.120  7.520  -0.731 | 0.905  < 0.001^***^ 0.465 | -0.064  0.021  -0.022 | 0.056  0.035  0.01 |
| Model_4 | RT ~ cong + synch +  (1 + cong * synch\| ID) + (1\| item) | congruency synchrony | 0.026  -0.013 | 0.006  0.011 | 65.412  64.412 | 4.445  -1.160 | < 0.001^***^ 0.25 | 0.014  -0.035 | 0.038  0.009 |

Level of significance: *: p≤ 0.05; **: p≤ 0.01; ***: p≤ 0.001

Abbreviations in alphabetical order: **b** – coefficient; **CI-l/CI-u** – lower and upper boundaries of the 95% confidence interval; **cong** – factor with two levels: congruent vs. incongruent; **df** – degrees of freedom; **IAT** – Implicit Association Test; **ID** – participants; **illusion** – Illusion score: difference of the illusion ratings (agency + ownership) between the synchronous and asynchronous condition; **item** – words presented during the IAT; **RT** – reaction times; **SE** – standard error **synch** – factor with two levels: synchronous vs. asynchronous.

**S5 Table. Model comparison for the analysis with linear mixed models of the BSRI-Data**

| **Model comparison** | **Full model** | **AIC** | **BIC** | **Chisq** | **Df** | **P** |
| --- | --- | --- | --- | --- | --- | --- |
| Model_1c | M_1c: BSRI ~ cong + (1 \| ID) + (1 \| item) | 8523.5 | 8547.2 |  |  |  |
| Model_2a vs. Model_1c | M_2a: BSRI ~ cong + (1\| ID) + (1\| item) | 8324.2 | 8353.8 | 201.31 | 1 | <0.001^***^ |
| Model_2b vs. Model_1c | M_2b: BSRI ~ synch + (1\| ID) + (1\| item) | 8527.2 | 8562.7 | 0.294 | 2 | 0.863 |
| Model_3a vs. Model_2a | M_3a: BSRI ~ cong + synch + (1\| ID) + (1\| item) | 8327.9 | 8369.3 | 0.3177 | 2 | 0.853 |
| Model_3b vs. Model_3a | M_3b: BSRI ~ cong * synch + (1\| ID) + (1\| item) | 8329.7 | 8382.9 | 2.204 | 2 | 0.332 |
| Model_3c vs. Model_3c(ref) | M_3b(ref): BSRI ~ cong * synch + (1\| ID) + (1\| item)  M_3c: BSRI ~ illusion*cong * synch + (1\| ID) + (1\| item) | 4120.1  4121.2 | 4156.5  4178.4 |  |  |  |
|  |  |  |  | 6.889 | 4 | 0.142 |

Level of significance: *: p≤ 0.05; **: p≤ 0.01; ***: p≤ 0.001

Abbreviations in alphabetical order: **AIC** – Akaike information criterion; **BIC** – Bayesian-Information-Criterion; **cong** – factor with two levels: congruent vs. incongruent; **df** – degrees of freedom; **IAT** – Implicit Association Test; **ID** – participants; **ill** – strength of the Enfacement Illusion (ownership + agency rating); **item** – words presented during the IAT; **BSRI** – revised version of the German BEM Sex-Role Inventory; **synch**

– factor with two levels: synchronous vs. asynchronous.

**S6 Table. Effect sizes in Confidence Intervals of BSRI-Data analysis with linear mixed models**

| **Model** | **Full model** | **Effect** | **b** | **SE** | **df** | **t** | **P** | **CI-l** | **Cl-u** |
| --- | --- | --- | --- | --- | --- | --- | --- | --- | --- |
| Model_1a | BSRI ~ 1 + (1 \| ID) | intercept | 3.917 | 0.074 | 67 | 53.02 | < 0.001^***^ | 3.771 | 4.062 |
| Model_1b | BSRI ~ 1 + (1 \| item) | intercept | 3.917 | 0.167 | 19 | 23.42 | < 0.001^***^ | 3.581 | 4.252 |
| Model_1c | BSRI ~ 1 + (1 \| item) + (1 \| ID) | intercept | 3.917 | 0.182 | 26.13 | 21.57 | < 0.001^***^ | 3.554 | 4.279 |
| Model_2a | BSRI ~ cong + (1 \| ID) + (1 \| item) | congruency | -0.591 | 0.041 | 2632.1 | -14.46 | < 0.001^***^ | -0.671 | -0.511 |
| Model_2b | BSRI ~ synch + (1 \| ID) + (1 \| item) | synchrony base | -0.004  0.021 | 0.057  0.052 | 2631  2631 | -0.074  0.396 | 0.941  0.692 | -0.122  -0.081 | 0.113  0.122 |
| Model 3a | BSRI~ cong + synch + (1 \| ID) + (1 \| item) | congruency synchrony base | -0.591  -0.004  0.021 | 0.041  0.057  0.05 | 2630  2630  2630 | -14.46  -0.076  0.412 | < 0.001^***^ 0.939  0.681 | -0.671  -0.118  -0.077 | -0.511  0.108  0.119 |
| Model_3b | BSRI ~ cong * synch + (1 \| ID) + (1 \| item) | congruency synchrony synch. * cong. | -0.571  -0.04  0.071 | 0.082  0.082  0.116 | 2628  2628  2628 | -6.989  -0.488  0.614 | < 0.001^***^ 0.625  0.539 | -0.731  -0.2  -0.155 | -0.411  0.12  0.3 |
| Model_3c | BSRI ~ ill * cong * synch + (1 \| ID) + (1 \| item) | illusion congruency synchrony synch*cong ill.*cong ill*synch ill*cong  *synch | 0.392  -0.041  0.382  -0.411  -0.523  -0.411  0.512 | 0.196  0.246  0.2870  0.243  0.229  0.243  0.316 | 1250.3  1267.3  1323.9  1325.6  1267.3  1325.6  1267.3 | 2.003  -0.167  1.329  -1.688  -2.280  -1.688  1.622 | 0.045^*^  0.087  0.184  0.092  0.023^*^  0.092  0.105 | 0.009  -0.522  -0.18  -1.181  -0.972  -0.887  -0.106 | 0.775  0.44  0.943  0.287  -0.074  0.065  1.13 |

Level of significance: *: p≤ 0.05; **: p≤ 0.01; ***: p≤ 0.001

Abbreviations in alphabetical order: **b** – coefficient; **CI-l/CI-u** – lower and upper boundaries of the 95% confidence interval; **cong** – factor with two levels: congruent vs. incongruent; **df** – degrees of freedom; **IAT** – Implicit Association Test; **ID** – participants; **ill** – strength of the Enfacement Illusion (ownership + agency rating); **item** – words presented during the IAT; ; **BSRI** – revised version of the German BEM Sex- Role Inventory; **SE** – standard error **synch** – factor with two levels: synchronous vs. asynchronous

# S2 Appendix. Supplementary Figures


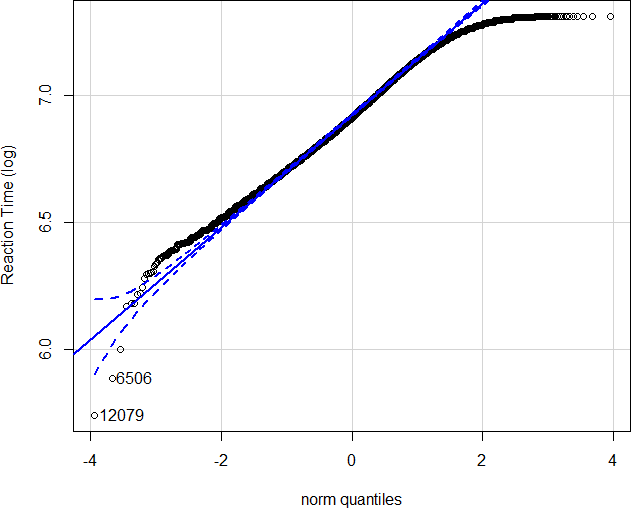


**S1 Fig. QQ-Plot of the log-transformed Reaction times***. Note:* visual inspection of the QQ-Plot of the log-transformed reaction times of the IAT indicated a normal distribution of the data.


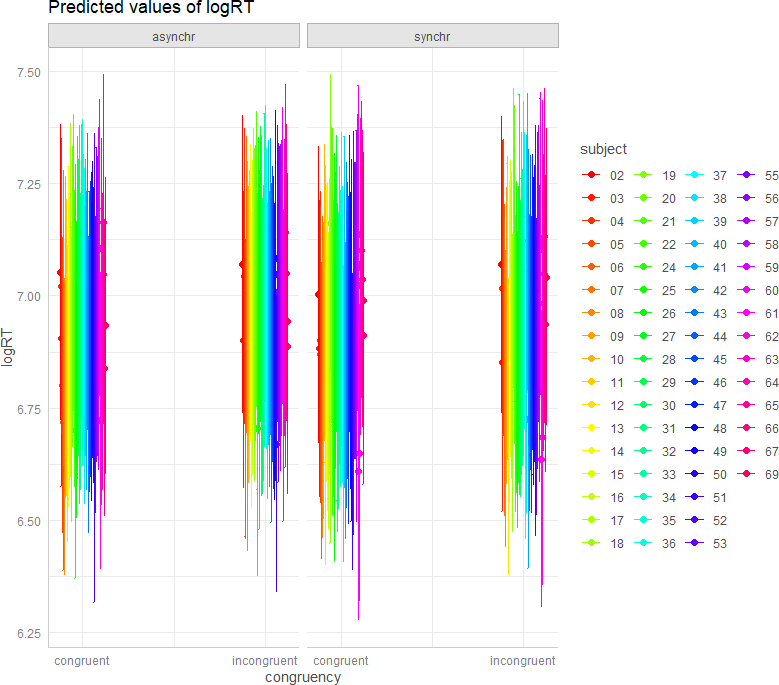


**S2 Fig. Predicted values of the log-transformed Reaction times**. Random coefficient Model_4:  *RT ~ congruency + synchrony + (1+congruency*synchrony| ID) + (1| item).* To investigate if slopes vary between the different subjects, a random-coefficient-model was fitted to the data. Comparing *Model_4* to *Model_3b* showed a significant difference for the model fit (*Model_3b* BIC: -7677.8; *Model_4* BIC: -8347.8). Consequently, allowing the slopes for the congruency and synchrony to vary explained significantly more additional variance (χ2(1) = 817.07, *p* < 0.001). S2 Fig. shows the predicted values of log RT and visualizes the random effects per subjects.

# S3 Appendix. Supplementary Analysis

# *S3.1. IAT-Data: D-score*

*S3.1.1. Data Preparation*

For comparability of the following results to the previous analysis with linear mixed models and in accordance with recommended steps for data preparation by Nosek et al. (2014), the raw data was preprocessed as described in *4.4.1*: Three participants were excluded due to low accuracy rate (<80%), trials with reaction time shorter than 200ms and longer than 1500ms were excluded and reaction times were log-transformed. After these steps the final sample consisted in 12,622 log transformed reaction times of 65 participants.

*S3.1.2. D-scores: Does the illusion of enfacing a male or female version of oneself affect implicit levels of gender identity representation?*

- - 1. *Is there a difference in mean reaction time between congruent and incongruent IAT blocks?*

A paired samples t-test was performed for the log-transformed mean reaction times to assess differences between congruent and incongruent IAT blocks. The distribution of differences between the congruent and incongruent group is not significantly different from normal distribution (Shapiro-Wilk normality test: *W* = 0.98033, *p* = 0.056). There was a significant difference between the congruent (*M* = 6.92, *SD* = 0.114) and the incongruent IAT block in mean log-transformed reaction times (*M* = 6.95, *SD* = 0.110), *t* (128) = -4,834, *p* < .001, *d* = 0.424, *CI* = [-0.039, -0.016])., see S3 Fig). Participants were faster in associating stimuli congruent with their self-identified gender.


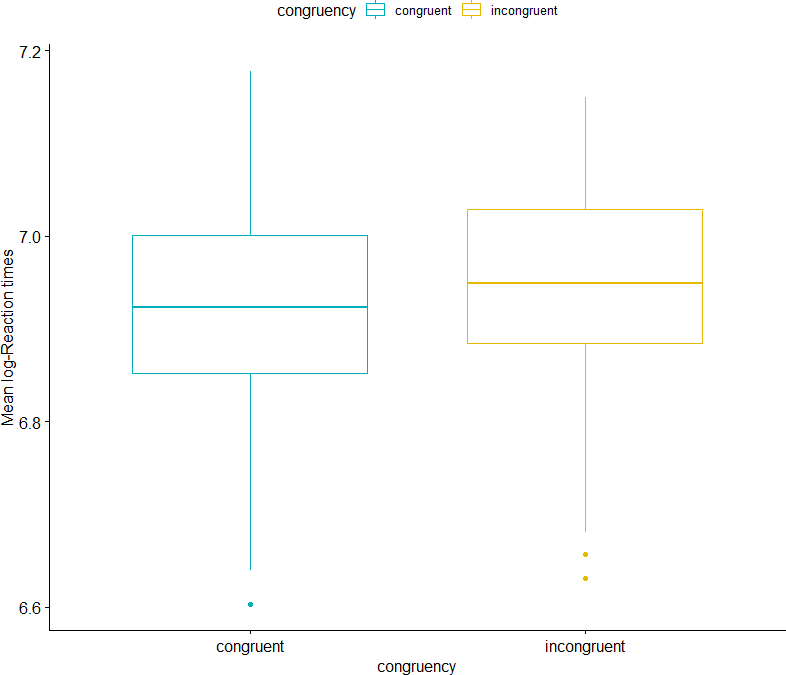


**S3 Fig.** **Boxplot of the log-transformed reaction times between IAT-blocks**. Boxplot of the per subject averaged log-transformed reaction times between IAT-blocks (congruent vs. incongruent): participants were significantly faster in the congruent IAT-blocks.

- - 1. *Is there a difference in D-scores between the synchronous and asynchronous condition?*

A 2x2-ANOVA was performed on the D-scores to assess differences between the synchronous and asynchronous condition. The D-score is computed as differences of the mean log-transformed reaction times between the incongruent and the congruent IAT blocks and divided by the standard deviation of mean log-transformed reaction times across conditions (Nosek et al., 2014). A higher D-score reflects faster reaction times in the congruent IAT blocks. The distribution of differences between the synchronous and asynchronous group is not significantly different from normal distribution (Shapiro-Wilk normality test: *W* = 0.975, *p* = 0.209). There was no significant main or interaction effect found: *gender*: *F*1, 126 = 1.187; *p* = 0.278, η^2^= 0.009, *synchrony*: *F*1, 126 = 0.902; *p* = 0.344, η^2^= 0.007; *interaction*: *F*1, 126 = 0.203;

*p* = 0.653, η^2^= 0.002 (see S4 Fig). This result complements the non-significant interaction of *congruency* and *synchrony* in *Model_3b*. The high standard deviations of the D-scores furthermore indicate high interindividual differences, which is demonstrated in the significant random-coefficient-model *Model_4*.


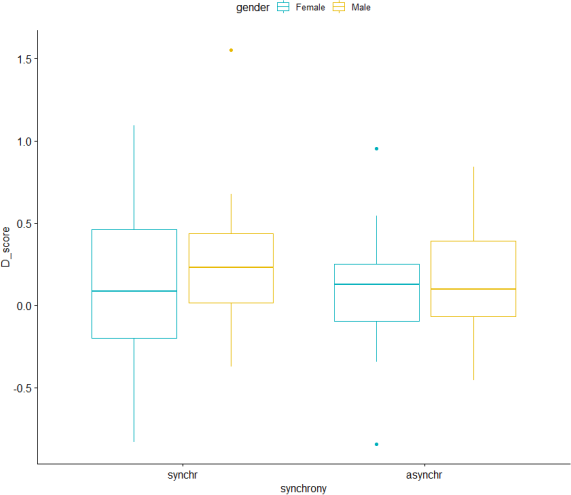


**S4 Fig**. **Boxplot of the D-scores with the factors’ synchrony and gender***.* Boxplot of the D-scores with the within-factor synchrony (asynchronous vs. synchronous) and the between-factor gender (female vs. male). There was no significant main or interaction effect found.

- - 1. *Does the strength of the Enfacement Illusion correlate with the D-Score?*

Tacikowski, Fust, and Ehrsson (2020) found that the strength of the enfacement illusion was related to a balancing of the implicit associations between the self and both genders. Based on

this finding a significant correlation of the strength of the enfacement illusion with the D-score was hypothesized. This correlation would reflect a similar effect: Subjects experiencing a stronger illusion of controlling and owning the face of another gender in the synchronous compared to the asynchronous condition (reflected by the illusion score) would show smaller D-scores (smaller congruency effects). The illusion score describes the difference of the illusion rating (agency + ownership) between the synchronous and asynchronous condition. The correlation of the illusion score and the D-score was not significantly different from 0 (Pearson’s product-moment correlation: *R* = 0.051, *t* = 0.403, *df* = 63, *p* = 0.688, *CI* = [-0.196, 0.291], see S5 Fig.). This non-significant correlation is in accordance with the non-significant results of *Model_3c* that assessed the interaction of the illusion score and the congruency effect as predictors in the linear mixed model.


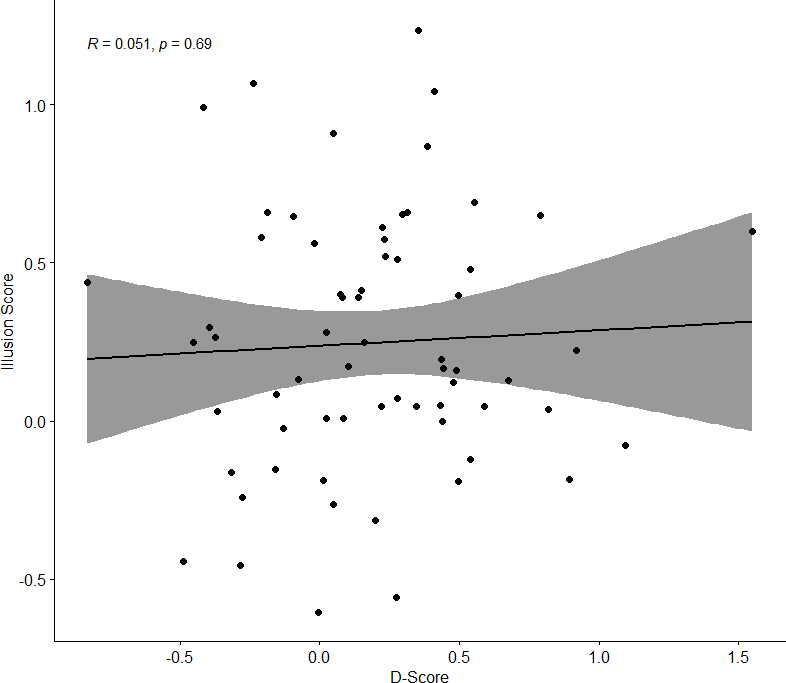


**S5 Fig**. **Correlation of the illusion score and the D-Score.** No significant correlation was found between these two variables.

*S3.1.3. Summary of the analysis of the gender identity IAT with D-score algorithm*

The above presented results confirm the previous analysis with linear mixed models. Firstly, the expected congruency effect was found comparing mean reaction times between congruent and incongruent IAT blocks: Participants were faster in associating stimuli congruent with their self-identified gender. Secondly, comparing the D-scores between the synchronous and asynchronous condition did not show a significant difference. Lastly, there was no correlation found between the Illusion scores and D-scores, indicating that the strength of the enfacement illusion was not related to ta balancing of implicit associations between the self and both genders as suggested in Tacikowski, Fust, and Ehrsson (2020).

# *S3.2 BSRI-DATA: Non-parametric ANOVA analysis*

*S3.2.1 Does the illusion of enfacing a male or female version of oneself affect the identification with stereotypical gender related traits (explicit level of gender identity)?*

A two-by-three ART-ANOVA with *congruency* and *synchrony* as a within factor was performed. The dependent variable was the rating of the BSRI-R. As expected, the main effect

of *congruency* was found to be significant (*F*1, 2647 = 147.442; *p* < 0.001, η^2^ = 0.053). No main effect of *synchrony* or interaction effect was found to be significant (*synchrony*: *F*2, 2647 = 0.086; *p* = 0.918, η^2^ = 0.00006; interaction: *F*2, 2647 = 0.54; *p* = 0.582, η^2^ = 0.0004; see S6 Fig*,*).


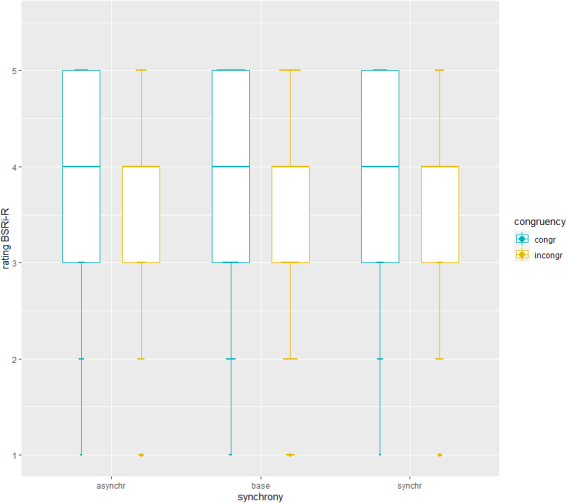


**S6 Fig.** **Boxplot of the ratings of BSRI-R with congruency and synchrony as factors**. Boxplot of the ratings of BSRI-R (range: 0-6, plot shows y-axis from 1-5.5) with *congruency* and *synchrony* as a within factor. In the corresponding ART-ANOVA a main effect of *congruency* was found to be significant, no main effect of *synchrony* or interaction effect was found.

*S3.2.2. Does the strength of illusion alter the rating of gender-stereotypes of personality?*

The correlation of the BSRI-R ratings and the illusion score was calculated separately for the synchronous and asynchronous condition. BSRI-R ratings were transformed in the way that high ratings for incongruent items are replaced by the corresponding low ratings. For example, when a male participant rates a conventionally female stereotype with 6 (highest rating) it was transformed to a value of 0 (lowest rating). The illusion score consists in the difference of the illusion rating in the synchronous and the asynchronous condition (synchronous-asynchronous). Both correlations of the illusion score and the BSRI-R in the synchronous and in the asynchronous condition were not significantly different from 0

(Pearson’s product-moment correlation: synch: *R* = 0.05, *t* = 1.305, df = 678, *p* = 0.192 and asynch: *R* = 0.071, *t* = 1.85, *df* = 678, *p* = 0.065).

*S3.2.3. Summary of the analysis of the BSRI-Data with ART-ANOVA*

These results confirm the analysis with linear mixed models of the BSRI-Data (see 4.5). Firstly, the expected congruency effect was found in the 2x3 ART-ANOVA: Participants rated items higher that matched their self-identified gender. Secondly, there was no main effect for synchrony found, demonstrating no significant difference in the rating of the BSRI-R between the synchronous and asynchronous condition. Lastly there was no correlation of the BSRI-R ratings and the illusion scores in the synchronous or asynchronous condition found, matching the results of the non-significant interaction of congruency*synchrony*illusion in *Model_3c* (see 4.5.2). This analysis does not support the hypothesis that a strong enfacement illusion balances the gender-related beliefs about the own personality.

# S4. Appendix. Study Material: Instructions and items with the corresponding German translation

# S4.1. Enfacement

Please, in the next section, keep your hand on your knees and focus on the screen. You will see your face with a filter overlayed. Move in a natural way in order to explore: blink, open your mouth and move your cheeks; and tilt your head towards up, down, left and right. When you are ready, press the spacebar.

*Bitte halten Sie während des nächsten Abschnitts Ihre Hände auf den Knien und konzentrieren Sie sich auf den Bildschirm. Sie werden Ihr Gesicht mit einem darübergelegten Filter sehen. Bewegen Sie sich möglichst natürlich, um diesen auszuprobieren: Blinzeln Sie, öffnen Sie Ihren Mund und bewegen Sie Ihre Wangen; bewegen Sie Ihren Kopf nach oben, unten, links und rechts. Wenn Sie bereit sind, drücken Sie die Leertaste.*

# S4.2. Gender Identity IAT

The test will start in a few seconds. Please listen to the instructions. Try to go as fast as possible while making as few mistakes as possible. If the word belongs to the categories FEMALE/MALE or SELF/OTHER, press LEFT. If the word does not belong to these categories, press right. When you are ready press the spacebar.

*Die Aufgabe beginnt in wenigen Sekunden. Bitte achten Sie auf die Instruktionen. Versuchen Sie so schnell wie möglich zu arbeiten und gleichzeitig möglichst wenige Fehler zu machen. Wenn das Wort zu den Kategorien „WEIBLICH/MÄNNLICH“ oder „SELBST/ANDERE“ gehört, klicken Sie links. Wenn das Wort nicht zu diesen Kategorien gehört, klicken Sie rechts. Wenn Sie bereit sind, drücken Sie die Leertaste.*

# S7 Table. Gender Identity IAT

| **Categories** | **Auditively presented words** |
| --- | --- |
| Selbst (*Self*) | Mir, selbst, mich, ich mein  *Me, self, myself, I, my* |
| Andere (*Other*) | Ihr, sie, ihnen, andere, ihre  *You, they, them, other, their* |
| Weiblich (*Female*) | Julia, Michaela, Anna, Laura, Sofie |
| Männlich (*Male*) | Johannes, Lukas, Daniel, Paul, Thomas |

*Note.*: participants assigned the heard words to one of the four categories which were presented on the screen.

# S8 Table. 10 items used from the revised BEM Sex-Role Inventory (BSRI-R).

| **Weiblich/*Female*** | **Männlich/*Male*** |
| --- | --- |
| bemüht sich, verletzte Gefühle zu besänftigen  *engaged to sooth hurt feelings* | hat Führungseigenschaften  *has leadership skills* |
| sinnlich  *sensual* | respekteinflößend  *authoritative* |
| empfindsam  *responsive* | verteidigt die eigene Meinung  *defends the own opinion* |
| herzlich  *sincere* | ist bereit, etwas zu riskieren  *willing to take risks* |
| zärtlich  *tender* | kraftvoll  *forceful* |
| fürsorglich  *caring* | selbstsicher  *confident* |
| an anderen Menschen interessiert  *interested in people* | entscheidungsfreudig  *decisive* |
| emotional  *emotional* | mächtig  *powerful* |
| sensibel | dominant |
| *sensitive* | *dominant* |

*Note.* Participants rated 10 items at baseline, 5 items in the synchronous, and 5 items in the asynchronous condition. English equivalents are presented in italic.


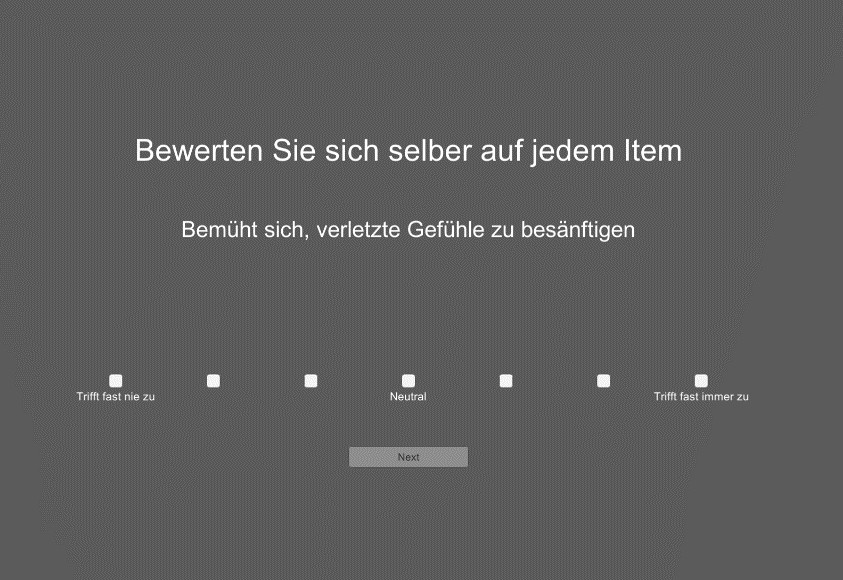


**S7 Fig.** **Example Item from the rating of the BSRI-R**


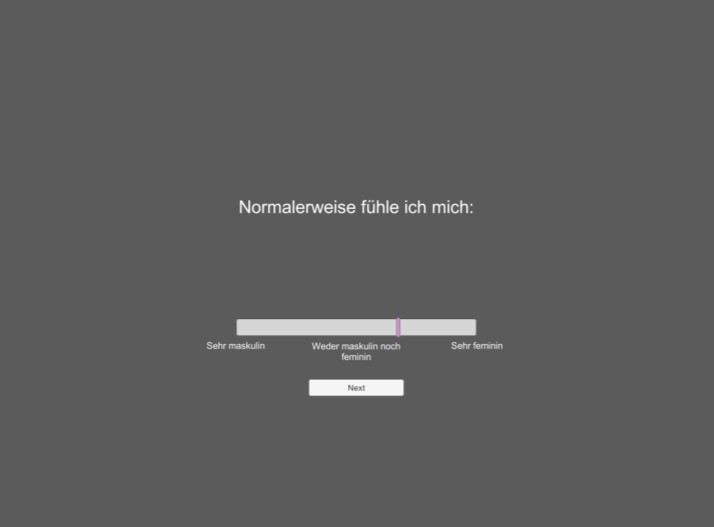


**S8 Fig**. **M-F-VAS rating at baseline**
