# Supplementary material for: Fluid face but not gender: Enfacement illusion through digital face filters does not affect gender identity
Source: PLoS One. 2024 Apr 3;19(4):e0295342. doi: 10.1371/journal.pone.0295342 (PMC10990241; doi:10.1371/journal.pone.0295342)
Supplement: S3 File — (PDF) [file pone.0295342.s003.pdf]

Request for Permission to Publish Content under CC-BY License

Dear Rights Holder or Representative,

I have submitted a paper for publication in a PLOS journal, and wish to include the content listed below in the paper. I'm hereby requesting your (or your company's or institution's) permission to include the content in my paper. Please note that all PLOS journals are published under a Creative Commons Attribution License (CC BY), which allows for unrestricted use and distribution, even commercial, as long as attribution is given to the creator or rights holder of the content. See https://creativecommons.org/licenses/by/4.0/.

To grant me permission to use the content in my PLOS paper, please fill in the information below and then scan the completed form and send it to me at my email address.

Thank you.

My name:

Maxwell Drimmer

My email address:

max.drimmer@gmail.com

Description of the content which I'm seeking permission to use (citation and/or title, and pasted screen shot, if applicable):

Figure 1. Overview of experimental procedure including the baseline measures, the enfacement phase, IAT and the post-manipulation measurements.
Figure 2. Depictions of My Twin Filter. (A1 and B1) Live captures of a woman's and man's face without any filters applied. (A2 and B2) Live captures of a woman's face with a male filter applied and a man's face with a female filter applied.

Link to the Content:

https://www.editorialmanager.com/pone/ViewPreviewImage.aspx?previewViewContext=1&sourceFileType=1&inventoryItemFileID=33449716
https://www.editorialmanager.com/pone/ViewPreviewImage.aspx?previewViewContext=1&sourceFileType=1&inventoryItemFileID=33449717

* * *

On behalf of myself or the rights holder, I hereby grant the permission sought herein.

Signature of Party Granting Permission:

MAX [illegible]

Date:

Aug 29 2023

Printed Name and Title:

Mr. Maxwell Drimmer
