# Supplementary material for: Fluid face but not gender: Enfacement illusion through digital face filters does not affect gender identity
Source: PLoS One. 2024 Apr 3;19(4):e0295342. doi: 10.1371/journal.pone.0295342 (PMC10990241; doi:10.1371/journal.pone.0295342)
Supplement: Attachment — Submitted filename: Response to Reviewers.docx [file pone.0295342.s004.docx]

**Response to Reviewers**

**Reviewer#1**

1. The study by Provenzano and colleagues investigated how the enfacement illusion induced with digital face filters affected gender identity. Participants observed their faces with a gender mismatched filter applied; their facial movements were synchronous (experimental) or asynchronous (control) with the movements they observed. Gender identity that was measured during and after the enfacement illusion with a set of implicit and explicit measures was not modulated.

The study addresses an interesting question, its design and analyses are correct, the methods and results are reported in full. I have several minor comments:

***We thank Reviewer#1 for the positive comments and evaluation of our work. We hope they will find our*** ***replies and the changes made to the manuscript to be satisfactory.***

1.1. Were all participants cisgender?

***We thank the reviewer for asking this question, as this is indeed an important point that needs to be clarified. Before the start of the actual enfacement procedure, all participants were asked to identify their gender as either masculine, feminine or non-binary. All participants identified themselves as cisgender, i.e., their self-identified gender matched their biological gender.***

***Considering all this, we added the following line to the manuscript (lines 112-113.)***

“All individuals who participated in the study described themselves as cisgender, i.e., their internal sense of gender corresponded to their biological sex identified at birth.”

1.2. Why was the gender identity IAT administered together with the enfacement illusion (participants still saw their filtered face during the IAT), rather than after it, as is commonly done in similar studies with embodiment of differently looking virtual bodies? If it was a replication of the experimental design used in the study by Tacikowski et al. (2020), could it have anyway contributed to the absence of significant effects in the IAT results? For example, a face, especially one considered one’s own, might be a much greater distractor compared to a body. This latter point (attention demands during the IAT+enfacement) is mentioned in the discussion but should it be expanded?

***We thank the reviewer for raising this interesting point. The reason we decided to administer the gender identity IAT while participants were still experiencing the enfacement illusion is twofold. On one hand, we wanted to replicate the experimental design used by Tacikowski and colleagues in their study (2020), which also used the auditory IAT measure during the experimental manipulation rather than after. On the other hand, by administering the IAT while participants were still experiencing the enfacement illusion we aimed at increasing the chances of significant findings on gender identity, considering that enfacement, as other embodiment illusions, might only show temporary effect on self-representation.***

***Although IAT should not be affected by general attention mechanism, as it relies on relative reaction times, it might indeed be possible that one’s own face could be a greater distractor than the body, As a matter of fact, own’s own face holds a special status in our cognitive and neural system, and body and face representation may have a different plasticity due to their different impact on self-identity. We already discussed this issue in the manuscript. Discussion section, lines 439-454 read as follow:***

“On the one hand, we focused on facial instead of body alterations. The face is arguably the most distinct visual characteristic of humans and is a core aspect of self-identity [16]. While some key attributes of faces, such as symmetry along the vertical axis, relatively fixed spatial relationships and social significance are also common to the body [47], others are arguably unique. Not only are body and face processing subject to different developmental periods (e.g., 44,45) and neural regions (e.g., 46,47) but, more importantly in this setting, one’s own body is constantly experienced from an egocentric frame of reference, while the face can only be observed from an allocentric frame of reference and in special conditions (e.g., a reflection in a mirror or screen). Such distinctions between facial and full-body processing - in particular the key role of the own face for self-identity - might be different enough to justify diverging findings between experimental manipulations of the full-body and the seen face. For instance, plenty of experimental evidence has shown that it is possible to experience embodiment illusion over full bodies even strongly different than one’s own and this in returns affects perception and attitudes towards the real body [51-53]. This is less clear for the face, and we speculate that could be a protective mechanism preserving one’s own self-identity that prevents the enfacement illusion to affect gender’s identity, i.e., body and face representation may have a different plasticity due to their different impact on self-identity.”

***Furthermore, we think that, as stated in the manuscript under “Limitations and outlook”, the cognitive resource required for performing the IAT might have distracted the participants from noticing the differences between the synchronous and asynchronous enfacement conditions, as anecdotally the experimenters noted that participants did not move their face (thus they did not experience a/synchronicity with the observed filtered face) while performing the IAT. . Thus, the IAT might have requested as many cognitive resources as to prevent participants from performing two concomitant tasks, i.e., moving their faces and responding as quickly as possible to the IAT stimuli. Thus, despite the IAT being preceded by 60 seconds of observation of synchronous or asynchronous facial movements, these participants might have experienced a similar perceptual condition while performing the IAT. This might have contributed to non-significant differences. To clean results from this possible confound, further studies should administer the IAT immediately after the a/synchronous conditions without vision of the filtered face. We added such indication in the manuscript as it follows (lines 506-508)***

In light of these factors, future studies should contemplate administering the IAT after the enfacement conditions and without the vision of the filtered face to mitigate these confounding effects.

1.3. Paragraph on Enfacement illusion through digital face filters repeats several statements from the introduction almost in the same words and does not refer to any of the results of the study. Would it be better if those arguments were presented in the context of the results?

***We thank the reviewer for highlighting the repetitions in the aforementioned paragraph. We have now reduced them. On the one hand, a certain degree of repetition is intended, as we wanted to stress, in our opinion, the importance and need of further investigation of these digital filters given the widespread availability and extensive usage. On the other hand, we agree that we did not add much compared to what was already present in the introduction. To rectify this, we added to the discussion section the following sentences (highlighted in yellow): (lines 401-414)***

“To achieve the enfacement illusion most studies have used either real people [8,15,16,38,39], images or videos of real people, self-other face morphs (14) or computer-generated avatars (18). In contrast to these stimuli and tools developed ad hoc by researchers and accessible only in a laboratory setting, we used widely accessible and popular social media face filters (Snap Inc., Santa Monica, CA). As expected, we show that a short period of active observation of filtered faces moving in synchrony induces subjective effects of the enfacement illusion. This suggests that millions of users might regularly go through short alterations of their bodily self-consciousness. It is important to remind that in our study participants were exposed to the digital face filters for only a brief period of time, whereas the general population’s exposure to these filters is much more prolonged and frequent, as the average usage of social media in most western countries is over 3 hours daily [40,41]. Moreover, on average 6 billion snaps, i.e., photos taken through Snapchat digital lens, are created by the user each day [42]. Thus, it might be that we missed illusory effects regarding gender identity that might occur frequently in daily life as a result of longer and repeated exposures to a stereotypically gender mismatched version of oneself.

Is also worth noting that whereas individuals who participated in our study were all young adults, in the general public social media are frequently used also by a younger adolescent population (97% of adolescents between the ages of 13 and 17 use at least one social media, 67% reported to use Snapchat; [23]), in a development period crucial to gender identification and characterized by the appearance of secondary sexual characteristics [43], where gender identity might be much more amenable to changes derived from exposure to stereotypically gender mismatched version of oneself.”

**Reviewer#2**

2. In the current manuscript, Provenzano and colleagues induced an enfacement illusion using a rendered version of the participants' own faces, altered to appear as the opposite gender. Based on a previous study using the full body illusion, authors investigated if the enfacement illusion could modulate not only their physical mental representation but also their gender identity. To this aim, authors compare an illusion synchronous condition, in which the modified face moved synchronously to the participants face, and an asynchronous control condition. Results showed a successful induction of the enfacement illusion in the synchronous condition according to subjective questionnaire ratings, reported agency and perceived similarity. However, authors found no modulation in gender identity, neither in explicit nor in implicit measures.

The manuscript is well-written, the hypotheses are clear, the statistical analyses are sound, and the discussion aligns with the main results. I only have a few minor concerns:

***We thank Reviewer#2, Dr. Mariano D’Angelo, for his positive comments about the present study. We hope they will find our answers to their minor concerns to be satisfactory.***

2.1. The details of the enfacement induction are not entirely clear to me. Did participants follow specific instructions for moving their head, eyes, and mouth? Or did they engage in free, exploratory movements? If the latter, it's possible that such free movements might have contributed to increase the variability between participants in the strength of the illusion. Additionally, could you provide information about the duration of the illusion induction?

***Before the actual enfacement phase, participants received verbal instructions from the experimenter on what to do and they were also presented with written instructions displayed on the PC monitor that informed them to move their face in a natural way. The instructions suggested the following movements: blinking, opening the mouth, moving the cheeks, tilting their head in the four possible directions (up/down & left/right). The instructions’ goal was to promote free and naturalistic exploratory movements in our participants in order to increase their sense of agency over the observed filtered face and thus strengthen the enfacement illusion. However, we agree that different degrees of facial activity between participants might have led to variations in the enfacement illusion's strength. We opted for free and naturalistic - instead of predefined and paced - facial movements illusion to preserve the ecological setting of the illusion and to not divert attention from the sensorimotor congruency with the observed filtered face. In order to render the procedure clearer, we added the following paragraph to the manuscript. Lines 155-159 reads as follow:***

“Before the enfacement procedure, participants were presented with written instruction that invited them to keep their hands on their knees and to explore the new filtered version of their face, presented on the screen in a mirror-like fashion, by performing natural movements such as: blinking, opening and closing their mouth, moving their cheeks, and tilting their head in all directions.”

***Regarding the duration of the enfacement illusion, both synchronous and asynchronous condition lasted 60 seconds each. We added this information in the enfacement phase paragraph as it follows (lines 167-168):***

The synchronous and asynchronous enfacement condition lasted 60 seconds each.

2.2. Another distinction from Tacikowski et al. is that the stimuli used in the enfacement illusion were derived from the participants' own faces. Consequently, "the other" in this study corresponded to a female/male "counterpart" of the participants, rather than an entirely distinct stranger. This aspect might have influenced the outcomes, as individuals may be more inclined to apply gender stereotypes to strangers. As a result, participants may not have merged self-other concepts as effectively, given that the "other" resembled more of a doppelganger than a completely unfamiliar individual onto whom stereotyped gender concepts could be projected.

***We thank Dr. D’Angelo for raising this interesting point, as it is indeed possible that the difference in body stimuli used by Tacikowski et al. (2020) and the filtered faces used in the present study might have led to the different results (please see also our reply to Point 1 of Reviewer 1). To this point, according to the predictive coding theory (Apps & Takiris, 2014) observing congruent spatio-temporal sensory information over another person’s face induces a conflict because the observed face does not match with the stored representation of the self-face (Porciello et al., 2018). In order to solve this conflict, the self-representation changes in accordance with the characteristics of the embodied “other body”, which might be the case of the study of Tacikowski et al (2020). It is possible, however, that in our study participants did not perceive the filtered face as a conflicting stimuli, and therefore the enfacement did not require any significant changes in the representation of the self. However, such hypothesis might only be tested with further studies where participants’ perception of the self-filtered face is investigated with ad-hoc explicit and implicit measures.***

***This possibility is now acknowledged in the “Limitations and Outlook” section of the manuscript (lines 517-525):***

Regarding the lack of clear changes in gender identity according to our measures, one potential explanation could be found the in absence of conflict between the observed layered faces and participants’ own faces. According to predictive coding theory [61], the presence of congruent spatio-temporal sensory information between one’s own face and another person’s face induces a conflict, as the observed face does not match the stored representation of the self-face [16]. To solve this conflict, self-representation may change in accordance with the characteristics of the enfaced “other face” or the embodied “other body”, as seen in the study by Tacikowski and colleagues [12]. It is possible, then, that in our study, participants did not perceive the filtered face as a conflicting stimulus, and as a result, enfacement did not necessitate significant changes in self-representation.

2.3. Related to the previous point, were participant aware that they were watching their counterpart in the video?

***Yes, participants were told in the instructions before the enfacement phase that they were about to “see their face with a filter overlayed”. This information is now present in the manuscript in the enfacement phase paragraph as it follows (line 159-160):***

Participants were informed that they were about to see their own faces with an overlay filter.

2.4. Authors could think to implement some Bayesian analysis to further strength their negative effect on gender identity changes.

***We thank Dr. D’Angelo for his suggestion, and we agreed on implementing Bayesian analysis to further strength the negative results on gender identity changes. We ran the analysis and added the following to the manuscript (lines 338-362)***

As absence of evidence does not equate to evidence of absence within the framework of frequentist statistics, we proceeded to enhance our analysis by employing Bayesian multilevel model to investigate the potential support for the null hypothesis (H0). To conduct these analyses, we utilized the R package "brms," which builds upon the rstan package. The application of Bayesian ANOVAs and multilevel models adhered to the methodology outlined in our previous works (Macauda et al., 2019, Ho et al., 2022). In this Bayesian context, posterior probability distributions were generated for the estimated parameters using non-informative priors. The Hamilton Monte Carlo sampling algorithm was employed to derive samples from the posterior distribution of each parameter. To ensure reliable results, we employed four separate Markov chains, each consisting of 1000 warm-up samples and an additional 2000 samples from the posterior distribution. The latter 2000 samples from each chain were retained for subsequent statistical analyses. Convergence of the chains' posterior distributions was verified by computing R-Hat statistics, all of which yielded values below 1.01. This indicated minimal variation between chains in comparison to within-chain variance. For the Bayesian multilevel models, we embraced the maximal random-effects structure that was suitable for our experimental design. This entailed incorporating random effects specific to each participant and condition.

Consistent with the frequentist analyses, the predictors were Gender, Condition and the interaction between the two. Two separate Bayesian models were fitted for BSRI Incongruent and BSI congruent. The 95% Bayesian credible intervals associated with these posterior distributions provided a credible range for the parameter, taking both the data and the model into account. Presence of an effect could be inferred if these credible intervals excluded the value of zero. Consistent with our frequentist counterparts, the Bayesian multilevel model indicated the absence of effects pertaining to Gender, Condition, and the interaction between Gender and Condition. This confirmation of the null hypothesis held true for both BSRI Congruent and BSI Incongruent conditions, as indicated by the crossing of the Credible Intervals over zero.

Finally, in the analysis of the Implicit Association Test, our Bayesian multilevel model, consistent with our frequentist analysis, also found no significant effects for Gender, Condition, or their interaction, as credible intervals span zero.
